# A protein interaction network centered on leucine-rich repeats and immunoglobulin-like domains 1 (LRIG1) regulates growth factor receptors

**DOI:** 10.1074/jbc.M117.807487

**Published:** 2018-01-09

**Authors:** Mahmood Faraz, Carl Herdenberg, Camilla Holmlund, Roger Henriksson, Håkan Hedman

**Affiliations:** From the Oncology Research Laboratory, Department of Radiation Sciences, Umeå University, SE-90187 Umeå, Sweden

**Keywords:** platelet-derived growth factor-C (PDGF-C), protein expression, protein-protein interaction, receptor tyrosine kinase, yeast two-hybrid, LRIG1, PDGFRA, PON2, PTPRK, ZBTB16

## Abstract

Leucine-rich repeats and immunoglobulin-like domains 1 (LRIG1) is a tumor suppressor and a negative regulator of several receptor tyrosine kinases. The molecular mechanisms by which LRIG1 mediates its tumor suppressor effects and regulates receptor tyrosine kinases remain incompletely understood. Here, we performed a yeast two-hybrid screen to identify novel LRIG1-interacting proteins and mined data from the BioPlex (biophysical interactions of ORFeome-based complexes) protein interaction data repository. The putative LRIG1 interactors identified in the screen were functionally evaluated using a triple co-transfection system in which HEK293 cells were co-transfected with platelet-derived growth factor receptor α, LRIG1, and shRNAs against the identified LRIG1 interactors. The effects of the shRNAs on the ability of LRIG1 to down-regulate platelet-derived growth factor receptor α expression were evaluated. On the basis of these results, we present an LRIG1 protein interaction network with many newly identified components. The network contains the apparently functionally important LRIG1-interacting proteins RAB4A, PON2, GAL3ST1, ZBTB16, LRIG2, CNPY3, HLA-DRA, GML, CNPY4, LRRC40, and LRIG3, together with GLRX3, PTPRK, and other proteins. *In silico* analyses of The Cancer Genome Atlas data sets revealed consistent correlations between the expression of the transcripts encoding LRIG1 and its interactors ZBTB16 and PTPRK and inverse correlations between the transcripts encoding LRIG1 and GLRX3. We further studied the LRIG1 function–promoting paraoxonase PON2 and found that it co-localized with LRIG1 in *LRIG1*-transfected cells. The proposed LRIG1 protein interaction network will provide leads for future studies aiming to understand the molecular functions of LRIG1 and the regulation of growth factor signaling.

## Introduction

Leucine-rich repeats and immunoglobulin-like domains 1 (LRIG1) is one of three LRIG paralogs in humans (1–3). LRIG1 has been shown to be expressed in all tissues analyzed to date (1, 4). Genetic deletion of *Lrig1* in mice causes hyperplasia in the skin (5, 6), lung (7), and intestines (8, 9) as well as duodenal adenomas (8). Thus, *Lrig1* is a *bona fide* tumor suppressor, as proposed in previous studies (1, 10). LRIG proteins are composed of a leucine-rich repeat domain, three immunoglobulin-like domains, a transmembrane domain, and a cytosolic tail. LRIG1 negatively regulates various oncogenic receptor tyrosine kinases (RTKs),^2^ including the epidermal growth factor receptor (EGFR) family members EGFR, ERBB2 (also called HER2), ERBB3, and ERBB4 (11, 12); hepatocyte growth factor receptor (MET) (13); rearranged during transfection (RET) (14); platelet-derived growth factor receptor α (PDGFRA) (15); and neurotrophic receptor tyrosine kinase 2 (NTRK2, also called TrkB) (16). Both ubiquitin ligase–dependent and ubiquitin ligase–independent mechanisms have been proposed as the molecular mechanism by which LRIG1 negatively regulates RTKs (for a review, see Ref. 17). Additionally, LRIG proteins may not exclusively regulate RTKs, LRIG2 has been shown to regulate ADAM proteases (18), and the sole LRIG homolog in *Caenorhabditis elegans*, Sma-10, positively regulates bone morphogenetic protein signaling (19).

In this study, we performed a yeast two-hybrid screen to identify novel LRIG1-interacting proteins and extracted high-confidence LRIG1 interactors from the BioPlex (biophysical interactions of ORFeome-based complexes) affinity-capture mass spectrometry–based protein interaction project (20, 21). The functional importance of these candidate LRIG1 interactors was assessed using shRNA-mediated down-regulation of protein expression followed by analyses of the effects on the LRIG1-induced down-regulation of PDGFRA. Our data reveal a functional LRIG1 protein interaction network comprising mostly novel and unanticipated components.

## Results

### Identification of LRIG1-interacting proteins

We performed a yeast two-hybrid screen of a human brain cDNA library using the cytosolic tail of LRIG1 as bait to identify putative novel LRIG1-interacting proteins. A screen of 2.3 × 10^6^ transformants yielded 10 bait-dependent clones. Of these bait-dependent clones, five were sequences that were not located in open reading frames and were therefore regarded as artifacts. The remaining five clones represented two different proteins: zinc finger and BTB domain–containing 16 (ZBTB16; three independent clones) and glutaredoxin 3 (GLRX3; two independent clones) (Table 1). We also used the general biological repository for interaction data sets (https://thebiogrid.org,^3^ May 4, 2017) to explore the BioPlex interaction data and identify additional LRIG1 interactors. Nine endogenous human embryonic kidney 293 (HEK293) T (HEK293T) cell line proteins (“hits”) that specifically co-purified with epitope-tagged LRIG1 and 10 epitope-tagged proteins (“baits”) that specifically interacted with endogenous LRIG1 were identified in the BioPlex data sets (20, 21) (Table 2). Paraoxonase 2 (PON2) interacted with LRIG1 both as a bait and a hit.

**Table 1:** **LRIG1-interacting proteins identified in the yeast two-hybrid screen**

Protein	Full name	Aliases	Reference sequence	Interacting amino acids*^a^*	Number of independent clones*^b^*	Effect on LRIG1 function*^c^*	Effect on LRIG1 level*^d^*
GLRX3	Glutaredoxin 3	TXNL2, PICOT	NP_001186797.1 (335 aa)*^e^*	322–335	2	0	0
ZBTB16	Zinc finger and BTB domain containing 16	PLZF, ZNF145	NP_001018011.1 (673 aa)	374–673	3	−	0

*^a^* Amino acid stretch in the protein representing the shortest isolated LRIG1-interacting yeast two-hybrid clone for the respective protein. Numbers refer to the numbering of the corresponding protein reference sequence. The total number of amino acids in the respective reference protein is indicated in parentheses.

*^b^* Number of independent clones obtained in the yeast two-hybrid screen. All five isolated cDNA clones had distinct nucleotide sequences.

*^c^* The effect on LRIG1 function was defined as positive or negative (−) when the corresponding shRNA yielded a significant increase or decrease, respectively, in the PDGFRA level upon co-transfection of HEK293 cells with *PDGFRA* and *LRIG1* in the triple co-transfection system.

*^d^* The effect on LRIG1 level was defined as positive or negative when the corresponding shRNA yielded a significant increase or decrease, respectively, of the LRIG1 level when HEK293 cells were co-transfected with *PDGFRA* and *LRIG1* in the triple co-transfection system.

*^e^* aa, amino acids.

**Table 2:** **LRIG1-interacting proteins extracted from the BioPlex network data sets**

Protein*^a^*	Full name	Role	Total number of bait interactions*^b^*	Effect on LRIG1 function*^c^*	Effect on LRIG1 level*^d^*
CANT1	Calcium-activated nucleotidase 1	Bait	8	0	0
CNPY3	Canopy FGF signaling regulator 3	Bait	9	−	0
CNPY4	Canopy FGF signaling regulator 4	Hit	9	−	−
GAL3ST1	Galactosyl-3-sulfonyltransferase-1	Bait	7	+	+
GML	Glycosylphosphatidylinositol-anchored molecule-like	Bait	24	−	−
HLA-DRA	Major histocompatibility complex, class II, DR α	Bait	29	−	−
HLA-E	Major histocompatibility complex, class I, E	Bait	44	0	0
LRIG2	Leucine-rich repeats and immunoglobulin-like domains 2	Hit	9	−	−
LRIG3	Leucine-rich repeats and immunoglobulin-like domains 3	Hit	9	−	−
LRRC40	Leucine-rich repeat containing 40	Hit	9	−	−
MTFR1L	Mitochondrial fission regulator 1-like	Hit	9	0	0
PON2	Paraoxonase 2	Bait/Hit	64/9	+	+
PTPRK	Receptor-like protein tyrosine phosphatase	Bait	88	0	0
RAB4A	RAB4A, member RAS oncogene family	Hit	9	0	+
SCARA3	Scavenger receptor class A member 3	Bait	40	0	0
SCRIB	Scribbled planar cell polarity protein	Hit	9	0	0
TUBB8	Tubulin β 8 class VIII	Hit	9	0	0

*^a^* High-confidence LRIG1-interacting proteins according to Ref. 20 and 21 data sets were retrieved from the BioGrid portal (https://thebiogrid.org,^3^ May 4, 2017).

*^b^* Total number of specific interactions of the respective bait in Refs. 20 and 21 combined.

*^c^* The effect on LRIG1 function was defined as positive (+) or negative (−) when the corresponding shRNA yielded a significant increase or decrease, respectively, in the PDGFRA level upon co-transfection of HEK293 cells with *PDGFRA* and *LRIG1* in the triple co-transfection system.

*^d^* The effect on the LRIG1 level was defined as positive (+) or negative (−) when the corresponding shRNA yielded a significant increase or decrease, respectively, in the LRIG1 level upon co-transfection of HEK293 cells with *PDGFRA* and *LRIG1* in the triple co-transfection system.

### Characterization of the co-transfection system

**Figure 1.**
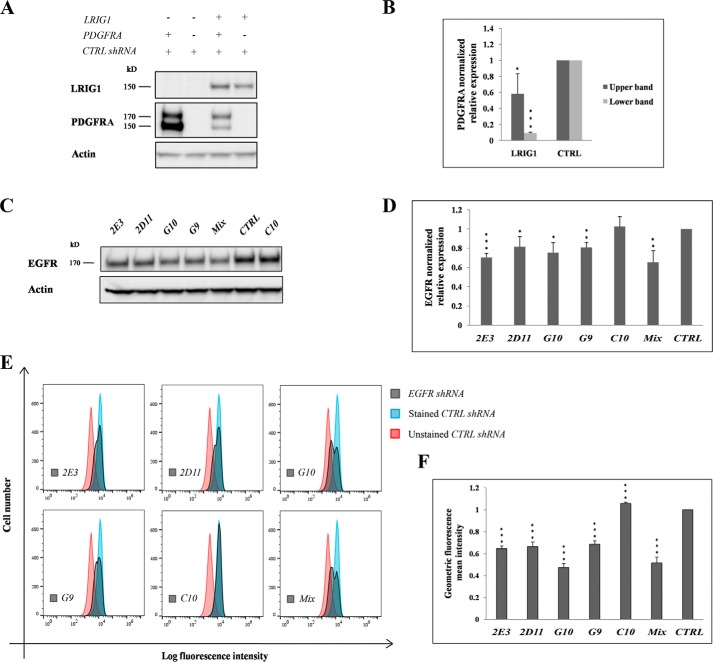
**Concomitant LRIG1-mediated down-regulation of PDGFRA expression and shRNA-mediated down-regulation of EGFR expression.** In the triple co-transfection system, HEK293 cells were transiently co-transfected with *LRIG1*, *PDGFRA*, and shRNA vectors. Four days after the transfection, cells were analyzed. *A*, representative Western blots showing the expression levels of LRIG1, PDGFRA, and actin in cells subjected to triple co-transfection with *LRIG1*, *PDGFRA*, and a non-targeting control shRNA. Actin was used as an internal loading control. *B*, quantification of three experimental replicates similar to the results shown in *A. C–F*, HEK293 cells were triple–co-transfected with *LRIG1*, *PDGFRA*, and one of five different EGFR shRNAs or an equimolar mixture of all five. Transfected cells were divided into two groups and analyzed using Western blotting (*C* and *D*) and flow cytometry (*E* and *F*). *C*, representative Western blot showing EGFR and actin levels in cells transfected with one of five different shRNAs, the shRNA mixture, or non-targeting control shRNA (*CTRL*). *D*, quantification of three biological replicates similar to the results shown in *C*. The apparent EGFR/actin ratio is shown. *E*, representative FACS histograms showing cell-surface EGFR staining. Unstained control sample (*red*) represents cells that were subjected to all experimental procedures, except the incubation with primary antibody. *F*, quantification of the specific EGFR staining intensity in three independent biological replicates, similar to the results shown in *E. Error bars*, S.D. Student's *t* test was used for the statistical analysis (*, *p* < 0.05; **, *p* < 0.01; ***, *p* < 0.001).

LRIG1 down-regulates PDGFRA expression when HEK293 cells are co-transfected with *LRIG1* and *PDGFRA* (15). Thus, we investigated the role of the identified LRIG1-interacting proteins in the LRIG1-induced down-regulation of PDGFRA to assess their functional importance. We devised a transient triple co-transfection system in which HEK293 cells were co-transfected with expression vectors encoding PDGFRA and LRIG1 together with shRNAs against the different LRIG1 interactors to achieve this goal. Because HEK293 cells express high, quantifiable levels of EGFR, we chose EGFR as the prototype shRNA target to optimize and validate the triple co-transfection system. Thus, by testing different transfection reagents, total amounts of plasmid DNA, and ratios of the included plasmids and by considering the resulting cell viability, the level of LRIG1-induced down-regulation of PDGFRA, and the level of shRNA target down-regulation efficiency, we finally arrived at the protocol described under “Experimental procedures.” Using this triple co-transfection protocol, LRIG1 was found to down-regulate the expression of the high- and low-molecular weight forms of PDGFRA, as reported previously (15), when cells were co-transfected with LRIG1 and a non-target control shRNA (Fig. 1, *A* and *B*). The 140 and 170 kDa bands are believed to represent the precursor and mature forms of PDGFRA, respectively (22). Five different shRNAs against EGFR, as well as the combination of all shRNAs, were evaluated to analyze the shRNA target down-regulation efficiency using this protocol. According to the Western blot analysis, four of the five individual shRNAs against EGFR, as well as the combination of all five, significantly down-regulated EGFR expression (Fig. 1, *C* and *D*). The strongest down-regulation was observed with the mixture, which induced a 35 ± 12% (S.D.; *n* = 3) down-regulation of EGFR expression compared with the level in the control (Fig. 1*D*). Flow cytometry was used to monitor the cell-surface expression of EGFR and evaluate the shRNA-mediated down-regulation of EGFR expression at the single-cell level. The four shRNAs that down-regulated EGFR expression on the Western blot, as well as the mixture, also down-regulated EGFR expression when cells were analyzed using flow cytometry (Fig. 1, *E* and *F*). The mean EGFR fluorescence intensity was down-regulated by 40.6 ± 5.5% (*n* = 3) by the shRNA mixture compared with the intensity of the non-target control shRNA. The histograms revealed double peaks for EGFR after shRNA-mediated down-regulation, indicating two distinct cell populations. In the case of the shRNA mixture, approximately half of the cells showed down-regulation of EGFR levels, whereas the other half expressed unaltered levels (Fig. 1*E*). The median specific fluorescence intensities of the two peaks were 24.8 ± 5.9% (*n* = 3) and 93.3 ± 27.7% (*n* = 3) of the control shRNA peak, respectively, indicating that approximately half of the cells exhibited an ∼75% decrease in EGFR levels. Thus, because the devised triple co-transfection system allowed us to simultaneously monitor LRIG1-mediated down-regulation of PDGFRA expression while the expression of a third protein was down-regulated with shRNA, we decided to use this experimental system to evaluate the functional importance of the LRIG1-interacting proteins.

### Effects of shRNAs targeting LRIG1 interactors on LRIG1-mediated down-regulation of PDGFRA expression

**Figure 2.**
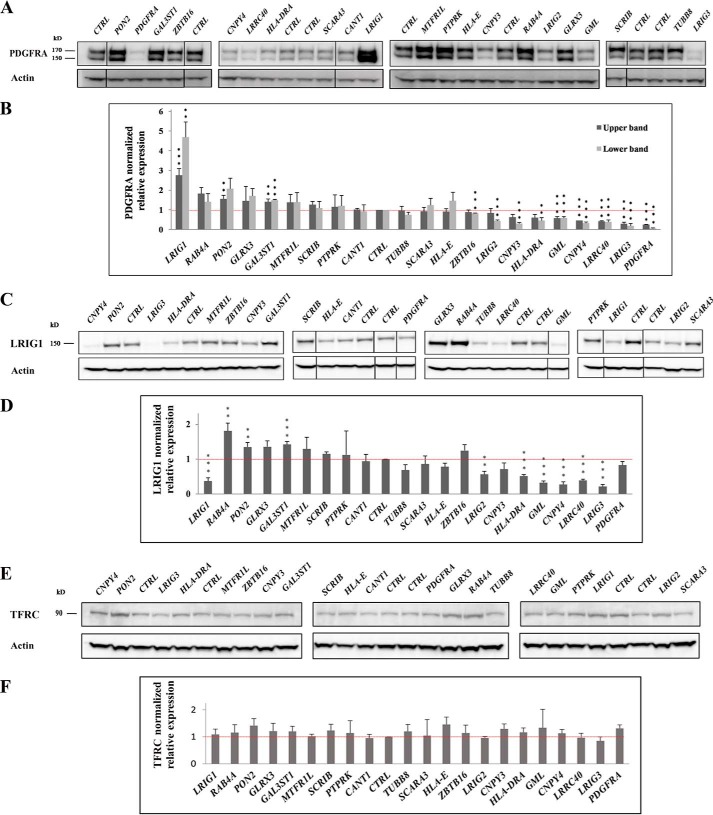
**LRIG1-mediated down-regulation of PDGFRA expression was modulated by shRNAs targeting LRIG1-interacting proteins.** In the triple co-transfection system, HEK293 cells were transiently co-transfected with *LRIG1*, *PDGFRA*, and specific shRNA mixtures as described in the legend to Fig. 1. *A*, representative Western blots showing PDGFRA and actin expression levels. *Vertical black lines*, non-adjacent lanes on the same blot. *B*, waterfall graph showing quantification of three experimental repeats (biological replicates), similar to the results shown in *A. C*, representative Western blots showing LRIG1 and actin expression levels. *Vertical black lines*, non-adjacent lanes on the same blot. *D*, quantification of three biological replicates, similar to the results shown in *C. E*, representative Western blots showing TFRC and actin expression levels. *F*, quantification of three biological replicates, similar to the results shown in *E. Error bars*, S.D. Student's *t* test was used for the statistical analyses, with a significance level of *p* < 0.01 (**, *p* < 0.01; ***, *p* < 0.001).

We used the triple co-transfection system to analyze the functional consequences of shRNA-mediated down-regulation of the different LRIG1 interactors. However, SCGB2A2 was excluded from these experiments because the expression of the corresponding mRNA is restricted (23), and we failed to detect any specific *SCGB2A2* transcripts in HEK293 cells using RT-PCR (data not shown). For each of the other interactors, five different shRNAs were mixed and used to co-transfect cells together with *LRIG1* and *PDGFRA*. Cells were transfected with each combination of plasmids three independent times by an investigator who was blinded to the order and identity of the plasmid mixtures. Four days after transfection, cells were lysed, and PDGFRA, LRIG1, and transferrin receptor (TFRC) levels were analyzed and quantified by Western blotting (Fig. 2). The mixtures of shRNAs targeting *PDGFRA* and *LRIG1* were included as controls; accordingly, co-transfection with the shRNA mixture targeting *PDGFRA* substantially decreased the levels of the PDGFRA protein (Fig. 2, *A* and *B*), whereas transfection with the shRNA mixture targeting *LRIG1* decreased LRIG1 levels (Fig. 2, *C* and *D*) and, as expected, increased PDGFRA levels (Fig. 2, *A* and *B*). Mixtures of shRNAs targeting two different LRIG1 interactors, PON2 and galactosyl-3-sulfonyl-transferase-1 (GAL3ST1), yielded a significant increase in the levels of one or both of the visible PDGFRA species, whereas shRNAs targeting eight other interactors, LRIG3, leucine-rich repeat containing 40 (LRRC40), canopy fibroblast growth factor (FGF) signaling regulator 4 (CNPY4), glycosylphosphatidylinositol anchored molecule-like (GML), major histocompatibility complex, class II, DR α (HLA-DRA), CNPY3, LRIG2, and ZBTB16, yielded a significant decrease in levels of one or both of the PDGFRA species (Fig. 2*B*). Overall, the effects of the different shRNA mixtures on LRIG1 levels were similar to the effects on PDGFRA levels (*i.e.* shRNA mixtures that increased or decreased PDGFRA levels tended to increase or decrease LRIG1 levels in a similar manner) (Fig. 2, *B versus D*), consistent with the finding that LRIG1 appears to be degraded together with its target RTK (12). Thus, LRIG1 expression was significantly up-regulated by shRNAs targeting RAB4A, PON2, and GAL3ST1 and significantly down-regulated by shRNAs targeting LRIG3, LRRC40, CNPY4, GML, HLA-DRA, and LRIG2. The level of the control membrane protein, TFRC, was not affected by any of the shRNA mixtures (Fig. 2, *E* and *F*).

### Correlations between the levels of LRIG1 and its interactors

**Figure 3.**
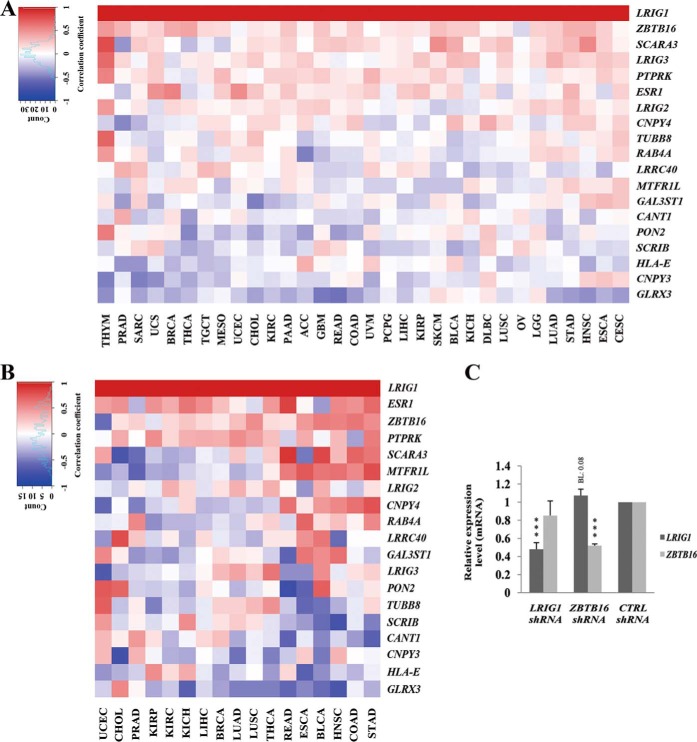
**Correlations between the expression of *LRIG1* and LRIG1-interactor mRNAs.** The expression of *LRIG1* and transcripts encoding LRIG1-interacting proteins was analyzed in the TCGA data set or in shRNA-transfected HEK293 cells. *A* and *B*, heat maps showing sorted correlation values for the LRIG1 interactor transcripts and *LRIG1* in cancer (*A*) or normal tissues (*B*). Correlations were calculated with Spearman's ρ using FPKM-UQ values from primary tumors or normal tissues. Correlation values were sorted by the mean correlation value for each gene, from the highest to the lowest. *C*, relative *LRIG1* and *ZBTB16* expression levels in HEK293 cells. HEK293 cells were transiently transfected with shRNA mixtures targeting *LRIG1* or *ZBTB16* or with a non-targeting control (*CTRL*) shRNA. Two days after transfection, total RNA was isolated, and *LRIG1*, *ZBTB16*, and *RN18S* expression levels were quantified using quantitative RT-PCR. The mean relative *LRIG1* and *ZBTB16* expression levels (*i.e.* the ratios between *LRIG1* or *ZBTB16* and *RN18S*) in four experiments are shown. *Error bars*, S.D. Student's *t* test was used for statistical analyses (***, *p* < 0.001). The tumor types and corresponding normal tissues included in the analysis were as follows: thymoma (*THYM*), prostate adenocarcinoma (*PRAD*), adrenocortical carcinoma (*ACC*), glioblastoma multiforme (*GBM*), rectal adenocarcinoma (*READ*), colon adenocarcinoma (*COAD*), thyroid carcinoma (*THCA*), testicular germ cell tumors (*TGCT*), mesothelioma (*MESO*), cholangiocarcinoma (*CHOL*), kidney renal clear cell carcinoma (*KIRC*), pancreatic adenocarcinoma (*PAAD*), uterine carcinosarcoma (*UCS*), breast invasive carcinoma (*BRCA*), uterine corpus endometrial carcinoma (*UCEC*), sarcoma (*SARC*), uveal melanoma (*UVM*), liver hepatocellular carcinoma (*LIHC*), renal kidney papillary cell carcinoma (*KIRP*), kidney chromophobe (*KICH*), lower-grade glioma (*LGG*), pheochromocytoma and paraganglioma (*PCPG*), lung squamous cell carcinoma (*LUSC*), ovarian serous cystadenocarcinoma (*OV*), lymphoid neoplasm diffuse large B-cell lymphoma (*DLBC*), skin cutaneous melanoma (*SKCM*), bladder urothelial carcinoma (*BLCA*), lung adenocarcinoma (*LUAD*), stomach adenocarcinoma (*STAD*), head and neck squamous cell carcinoma (*HNSC*), esophageal carcinoma (*ESCA*), and cervical squamous cell carcinoma and endocervical adenocarcinoma (*CESC*).

Consistent co-expression of genes may indicate that they have a shared function. We investigated possible correlations between the expression of the transcripts encoding the respective LRIG1 interactor and *LRIG1* in The Cancer Genome Atlas (TCGA) data sets to determine whether the expression of any of the LRIG1 interactors was associated with LRIG1 expression. *ESR1*, encoding estrogen receptor 1, was included as a control because estrogen signaling is known to positively regulate *LRIG1* expression (24). Accordingly, the expression levels of *ESR1* and *LRIG1* were correlated in breast cancer (Spearman's correlation coefficient, 0.56), other cancers (Fig. 3*A*), and many normal tissues (Fig. 3*B*). Among the LRIG1 interactors, *ZBTB16* and receptor-like protein tyrosine phosphatase (*PTPRK*) showed moderate but consistent positive correlations with *LRIG1* expression in both cancer tissues and normal tissues, whereas *GLRX3* showed a moderate but consistent negative correlation with *LRIG1* expression in both cancer and normal tissues. The other LRIG1 interactors showed more mixed correlation patterns, with both positive and negative correlations, as well as no correlations, with *LRIG1* expression in the different cancer and normal tissue types. Because ZBTB16 functions as a transcriptional regulator (for a review, see Ref. 25), we tested the hypothesis that ZBTB16 regulates *LRIG1* transcription in HEK293 cells (Fig. 3*C*). We simultaneously tested the complementary hypothesis that LRIG1 regulates the transcription of *ZBTB16*. Although a trend toward *LRIG1* up-regulation was observed in response to the shRNA-mediated down-regulation of *ZBTB16* expression, this trend was not significant (*p* = 0.08), and the data did not support the hypothesis that ZBTB16 positively regulates *LRIG1* expression.

### Effects of individual shRNAs targeting RAB4A and PON2

**Figure 4.**
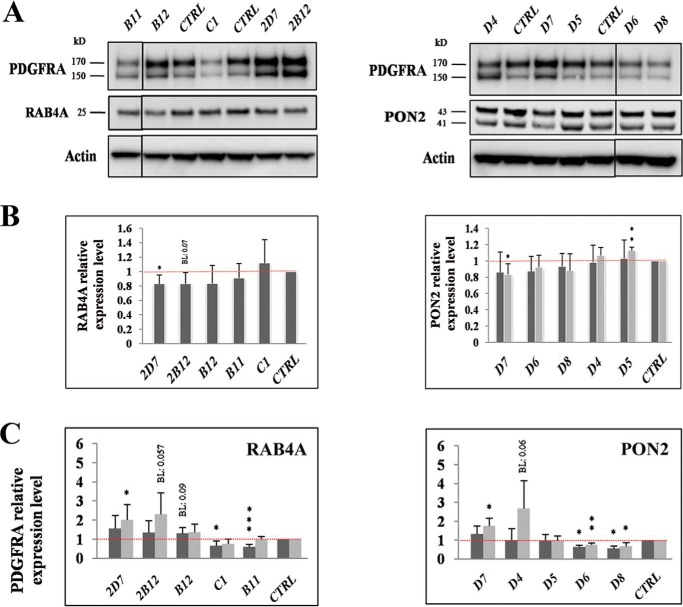
**PON2 and RAB4A promoted LRIG1-mediated down-regulation of PDGFRA expression.** In the triple co-transfection system, cells were co-transfected with *LRIG1*, *PDGFRA*, and individual shRNA vectors targeting the LRIG1 interactors RAB4A and PON2 as described in the legend to Fig. 1. *A*, representative Western blots showing RAB4A, PON2, and PDGFRA expression in HEK293 cells triple–co-transfected with *LRIG1*, *PDGFRA*, and different shRNAs targeting *RAB4A* (*left*) or *PON2* (*right*). *Vertical black lines*, non-adjacent lanes on the same blot. *B*, quantification of RAB4A (*left*) and PON2 (*right*) protein levels after triple co-transfections with *LRIG1*, *PDGFRA*, and the indicated shRNAs. The results from four independent experiments similar to *A* are shown. *C*, quantification of PDGFRA protein levels from four biological replicates as described in *B. Darker* and *lighter gray bars* represent the upper and lower PDGFRA bands, respectively. Statistical analyses were performed as described in the legend to Fig. 1. *Error bars*, S.D.

Next, we wanted to determine how well the individual shRNAs were able to down-regulate the levels of the corresponding protein and to what extent this effect correlated with the effects on PDGFRA levels. Therefore, we focused on the proteins that appeared to promote LRIG1 function or expression: RAB4A, PON2, and GAL3ST1. First, we screened a series of commercially available antibodies for their ability to detect endogenous RAB4A, PON2, and GAL3ST1 in HEK293 cells using Western blotting. We identified antibodies that specifically recognized endogenous RAB4A and PON2 (Fig. 4*A*); however, no antibodies specifically recognized endogenous GAL3ST1 (data not shown). The levels of the RAB4A and PON2 target proteins were decreased to a relatively modest extent, ∼20%, by the most efficient shRNAs (Fig. 4*B*). However, intriguingly, shRNAs that decreased the levels of the target protein to the greatest extent (2D7 for RAB4A and D7 for PON2) also up-regulated PDGFRA expression to the greatest extent in the triple co-transfection system (Fig. 4*C*). In fact, for RAB4A, we observed a perfect match between the down-regulation of the three best protein targets (2D7, 2B12, and B12) and the corresponding up-regulation of PDGFRA expression (Fig. 4, *B* and *C*). Based on these results, the down-regulation of the protein target, at least for RAB4 and PON2, was responsible for the observed effects of the shRNA on PDGFRA levels.

### Subcellular localization of PON2

**Figure 5.**
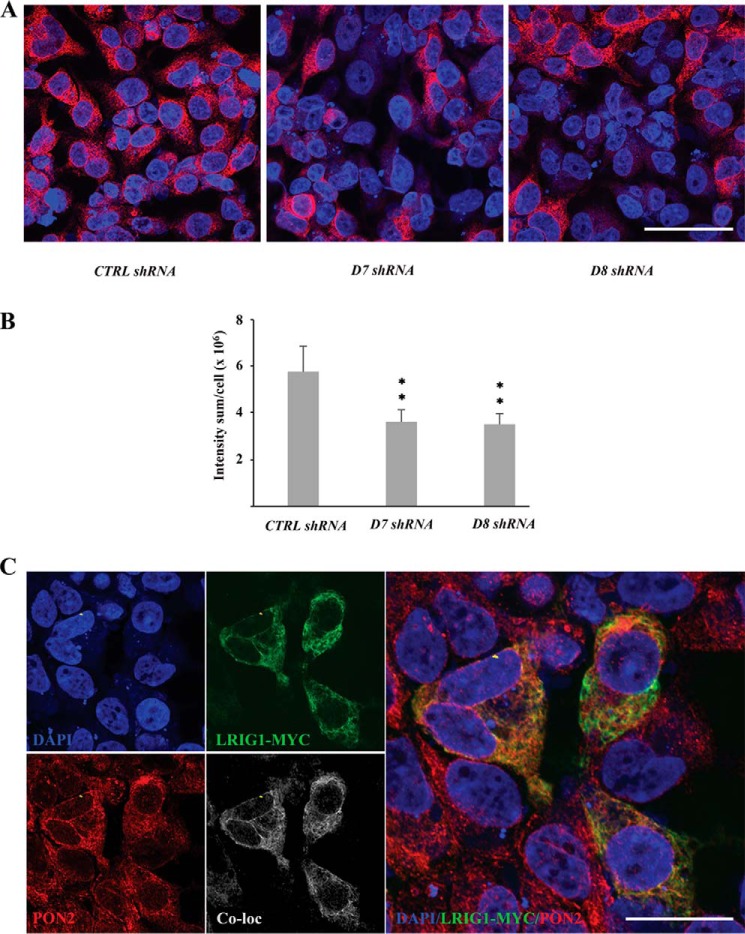
**Subcellular localization of PON2 and LRIG1 in transfected HEK293 cells.** HEK293 cells were triple–co-transfected with *LRIG1*, *PDGFRA*, and shRNA targeting *PON2* or control shRNA; stained with antibodies against PON2 and LRIG1; and then analyzed using laser confocal microscopy. *A*, PON2 staining of cells transfected with the non-target control shRNA (*left*), shRNA D7 targeting *PON2* (*middle*), or shRNA D8 targeting *PON2* (*right*). *B*, quantifications of four experiments similar to *A. Error bars*, S.D. **, *p* < 0.01. *C*, HEK293 cells were triple–co-transfected with *LRIG1*, *PDGFRA*, and non-target control shRNA and stained with DAPI and antibodies against Myc (LRIG1) and PON2. *Top left*, DAPI staining of cell nuclei (*blue*). *Bottom left*, PON2 staining (*red*). *Top middle*, LRIG1 (Myc) staining (*green*). *Bottom middle*, LRIG1 and PON2 co-localization (*white*). *Right*, overlay of DAPI, LRIG1, and PON2 staining. *Scale bar*, 10 μm.

Next, we aimed to investigate the subcellular localization of RAB4A and PON2 using immunofluorescence staining and laser confocal microscopy. However, the RAB4A antibody used for Western blotting did not specifically recognize RAB4A in immunofluorescence staining, nor did other tested RAB4A antibodies (data not shown). Thus, we were unable to analyze endogenous RAB4A expression in HEK293 cells using immunofluorescence staining and confocal microscopy. In contrast, the PON2 antibody used for Western blotting also specifically recognized the endogenous protein in immunofluorescence staining (Fig. 5, *A* and *B*). Thus, shRNAs D7 and D8 were found to down-regulate the fluorescence intensity by 37.6 and 39.1%, respectively, revealing the specificity of the PON2 antibody used in the present study. PON2-specific immunoreactivity displayed a punctate distribution and was predominantly localized in the cytoplasm of HEK293 cells (Fig. 5, *A* and *C*). Some, but not all, of the LRIG1- and PON2-positive spots and pixels co-localized with each other (Fig. 5*C*). A spatial correlation analysis of the pixel intensity in 12 LRIG1-transfected cells revealed a Pearson's correlation coefficient for LRIG1 and PON2 co-localization of 0.8224.

### Effects of PON2 and ZBTB16 down-regulation on LRIG1-null cells

**Figure 6.**
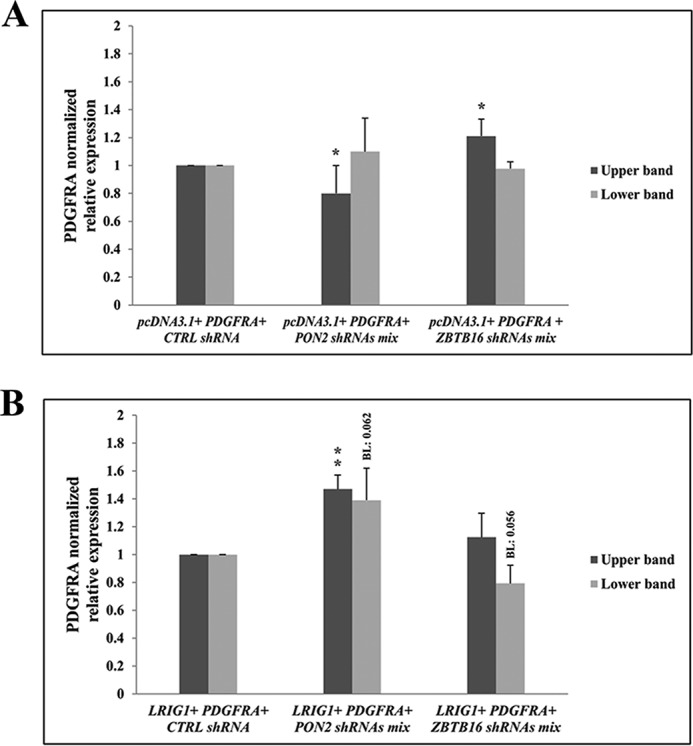
**Effects of PON2 and ZBTB16 down-regulation on LRIG1-deficient cells.** LRIG1-null HEK293T cells were transiently triple-transfected with the *pcDNA3.1* control vector (*A*) or *LRIG1* (*B*) together with *PDGFRA* and one of the following shRNA mixtures: non-targeting shRNAs (*CTRL*), shRNAs targeting *PON2*, or shRNAs targeting *ZBTB16*. The relative PDGFRA levels observed 4 days after transfection by Western blotting are shown; actin served as the reference protein. The experiment was repeated four independent times. *Error bars*, S.D. Student's *t* test was used for statistical analyses (*, *p* < 0.05; **, *p* < 0.01).

In principle, the observed up- and down-regulation of PDGFRA expression in the triple co-transfection system was caused either by effects on the PDGFRA–down-regulating function of LRIG1 or, alternatively, by other PDGFRA expression-modulating effects that are independent of LRIG1. We generated LRIG1-null HEK293T cells to investigate whether the down-regulation of PON2 and the yeast two-hybrid prey ZBTB16 modulated PDGFRA expression through an LRIG1-dependent or -independent mechanism. In these cells, shRNA-induced down-regulation of PON2 expression did not induce an up-regulation of PDGFRA expression (Fig. 6*A*); instead, PDGFRA expression was down-regulated. Similarly, the shRNA-induced down-regulation of ZBTB16 in LRIG1-null cells did not induce the down-regulation of PDGFRA expression (Fig. 6*A*); instead, PDGFRA expression was up-regulated. However, when the LRIG1-null cells were transfected with LRIG1, the shRNA mediated down-regulation of PON2, and ZBTB16 induced up-regulation and down-regulation (borderline significance, *p* = 0.056) of PDGFRA expression, respectively, similar to the findings observed in wildtype HEK293 cells (Fig. 6*B*). Thus, the PDGFRA expression-modulating effects of PON2 and ZBTB16 down-regulation depend on LRIG1.

## Discussion

**Figure 7.**
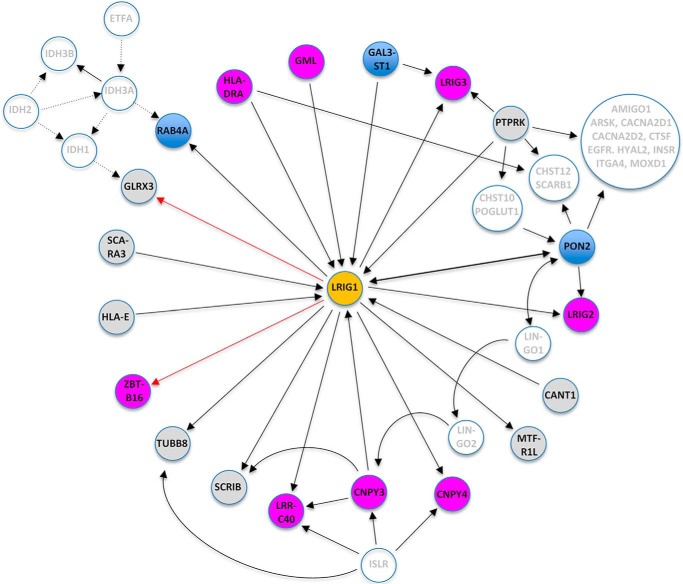
**Hypothetical LRIG1 regulatory network.** Shown is a schematic and hypothetical regulatory network based on the yeast two-hybrid screen results, the BioPlex protein interaction data, and the functional evaluations in the present study. Proteins are illustrated by *circles*, and protein interactions are indicated by *solid* or *dotted lines. Arrows* point in the corresponding direction of interaction (*i.e.* from bait to prey in the case of interactions discovered by the yeast two-hybrid experiments (*red arrows*) or from bait to hit protein in the case of the BioPlex affinity-purification mass spectrometry data (20, 21) (*black solid arrows*)). *Dotted lines*, interactions described in other high-throughput screens (49–51). The *colors* of the *circles* indicate the functionality of the respective protein in our PDGFRA assay; *blue circles* depict proteins that appeared to promote the PDGFRA–down-regulating function of LRIG1 or the LRIG1 level itself; *magenta circles* depict proteins that appeared to suppress the PDGFRA–down-regulating function of LRIG1; *gray circles* depict proteins that appeared to neither promote nor suppress the PDGFRA–down-regulating function of LRIG1; and *white circles* depict proteins that did not directly interact with LRIG1, and therefore, their functions were not evaluated in the current study.

In this study, we identified a series of novel LRIG1-interacting proteins and evaluated their importance for LRIG1 function. We assessed functionality by monitoring the levels of the PDGFRA protein after triple co-transfections of HEK293 or HEK293T cells with *LRIG1*, *PDGFRA*, and shRNAs against the different LRIG1 interactors. We chose to use shRNA instead of siRNA because shRNA induces fewer off-target effects than siRNA (26); we also used a mixture of five different shRNAs against each of the LRIG1 interactors instead of single shRNAs for additional reasons. First, in our optimization experiments, the mixture of shRNAs targeting EGFR was more effective than any of the individual shRNAs. Second, because many of the intended targets are known to be expressed as multiple splice variants, a mixture of different shRNAs is preferred to reduce the risk of failure in targeting important alternatively spliced transcripts. None of the nine non-LRIG proteins identified as being functionally important had previously been implicated as a determinant of LRIG1 function. Thus, we believe that our suggested functional LRIG1 protein interaction network (Fig. 7), which includes several previously unanticipated LRIG1 modulators, will provide a novel map for future studies aimed at understanding the molecular functions of the LRIG proteins.

Our yeast two-hybrid screen revealed two potential and novel LRIG1 interactors, ZBTB16 and GLRX3; the former, but not the latter, appeared to inhibit the LRIG1-mediated down-regulation of PDGFRA expression. ZBTB16 is a transcriptional regulator (25) that was also recently shown to play a role in protein ubiquitination and degradation (27). The expression of *ZBTB16* and *LRIG1* was consistently correlated in TCGA data sets, suggesting that the two genes may have a shared function. Additionally, because ZBTB16 is a transcriptional regulator, we investigated whether *LRIG1* expression was directly regulated by ZBTB16. However, this hypothesis was not validated, as the shRNA-induced down-regulation of *ZBTB16* expression did not yield a concomitant down-regulation of *LRIG1* expression. Intriguingly, single nucleotide polymorphisms in both the *LRIG1* and *ZBTB16* genes are associated with glioma susceptibility (28, 29). The relationship between the physical ZBTB16-LRIG1 interaction and the functions of LRIG1 and ZBTB16 in glioma susceptibility remains an important area for future research. The shRNA mixture targeting GLRX3 did not modulate the PDGFRA–down-regulating function of LRIG1, which may or may not reflect technical shortcomings, alternative functions, or a truly non-functional interaction, as discussed below.

In the BioPlex data sets, LRIG1 physically interacted with both LRIG2 and LRIG3. As shown in the study by Rafidi *et al.* (30), LRIG3 interacts with LRIG1 and opposes the ERBB-regulating function of LRIG1, consistent with the results of the current study. Intriguingly, LRIG2 also appeared to stabilize PDGFRA in LRIG1-overexpressing HEK293 cells. Thus, LRIG1, LRIG2, and LRIG3 all appear to participate in a regulatory subnetwork through physical interactions, and LRIG2 and LRIG3 oppose the RTK-restraining function of LRIG1.

In fact, the three LRIG proteins appear to be part of a functionally important cluster of interactors in our envisioned LRIG1 protein interaction network (Fig. 7). In addition to LRIG1, LRIG2, and LRIG3, this cluster, or subnetwork, comprises the LRIG1 interactors GAL3ST1, PTPRK, and PON2 as well as a large group of proteins that interacted with both PTPRK and PON2 but did not directly interact with LRIG1. Of these LRIG1 interactors, PON2 showed several particularly intriguing features. First, as described above, PON2 was a determinant of the PDGFRA–down-regulating function of LRIG1. Notably, shRNAs targeting *PON2* that down-regulated the PON2 expression also up-regulated PDGFRA expression in cells co-transfected with LRIG1. In contrast, when PON2 expression was down-regulated in LRIG1-null cells, PDGFRA expression was not up-regulated. This experiment unequivocally showed that the effect of PON2 on promoting PDGFRA expression in HEK293T cells depended on LRIG1. Second, PON2 interacted bidirectionally with LRIG1 in the BioPlex experiments. Third, in the BioPlex experiments, PON2 interacted with both LRIG1 and LRIG2, which also may indicate a physiologically relevant interaction because a protein may be less likely to interact with both LRIG1 and LRIG2 only by chance. Fourth, PON2 (together with PTPRK) interacted with EGFR, which was the only LRIG1 interaction network protein described here that was anticipated based on previously published work. Fifth and finally, PON2 also bidirectionally interacted with LINGO1, another leucine-rich repeat and immunoglobulin-like domain–containing protein that has been reported to negatively regulate TRK (NTRK) receptors in a manner that appears to be highly similar to the mechanism described for LRIG1 (31). PON2 is a type II transmembrane protein (32) with lactonase enzymatic activity (33) that has been reported to protect the plasma membrane from lipid peroxidation, regulate its glucosylceramide levels (32), and facilitate the function of mitochondria (34). PON2 localizes to the nuclear membrane (35), endoplasmic reticulum (35), mitochondria (34), and plasma membrane (32). Accordingly, we observed a punctate distribution and extensive co-localization of PON2 and LRIG1 in the cytoplasm of HEK293 cells. Whether this co-distribution corresponds to any or several of the aforementioned PON2 locations remains to be investigated. Additionally, PON2 was recently shown to promote pancreatic tumor growth by regulating glucose transporter 1–mediated glucose transport (36). Investigations of the possible causal relationships between all of these molecular functions, clinical associations, and the LRIG1-PON2 interaction reported in the present study will be important. PTPRK shared 15 interactors with PON2. However, PON2 also interacted with LRIG2, but PTPRK instead interacted with LRIG3. In contrast with PON2, PTPRK was not identified as functionally important in our experimental paradigm. Nevertheless, PTPRK has been reported to negatively regulate EGFR-mediated signaling through dephosphorylation of the receptor (37, 38). Additionally, *PTPRK* and *LRIG1* expression was consistently correlated in a wide range of cancer types and normal tissues. Based on these data, PTPRK may play an important role in the RTK-suppressing functions of LRIG1. GAL3ST1 appeared to support the function of LRIG1 and interacted both with LRIG1 and LRIG3. GAL3ST1 is a galactosylceramide sulfotransferase that catalyzes the sulfonation of membrane glycolipids, generating sulfatides (39). Notably, three of the additional 14 common PON2-PTPRK interactors were also sulfotransferases (CHST10 and CHST12) or sulfatases (ARSK). The possible role of glycolipid or glycoprotein sulfate groups in the function of LRIG1 is intriguing and requires further investigation.

Another apparent cluster of LRIG1 interactors included LRRC40, CNPY3, and CNPY4 plus ISLR, which interacted with tubulin β8 class VIII (TUBB8), LRRC40, CNPY3, and CNPY4 but did not directly interact with LRIG1. LRRC40, CNPY3, and CNPY4 all seemed to oppose the PDGFRA–down-regulating function of LRIG1. LRRC40 is a cytoplasmic or nuclear protein that also contains a leucine-rich repeat domain but no immunoglobulin-like or transmembrane domains. To the best of our knowledge, a clear function has not been attributed to LRRC40. CNPY3 and CNPY4 belong to a family of structurally related proteins that also includes the zebrafish FGF–signaling promoter CNPY1 (40, 41). CNPY3 is a co-chaperone that, together with HSP90B1, is required for the folding of multiple TOLL-like receptors (42). Less is known about CNPY4; however, its structural similarity to CNPY3 suggests that it might also function as a (co-)chaperone. Thus, CNPY3 and CNPY4 may be (co-)chaperones that are required for the folding of PDGFRA and LRIG1 (and possibly also scribbled planar cell polarity protein (SCRIB), LRRC40, and ISLR), rather than being regulators of the function of LRIG1 *per se*.

RAB4A supported the function of LRIG1 in HEK293 cells. RAB4A is involved in the recycling of early endosomes (43) and has been implicated as an important determinant of the invasiveness of cancer cells (44), thereby providing a possible molecular link between LRIG1 and its apparent metastasis-suppressing function (45–48). Intriguingly, both GLRX3 and RAB4A have been reported to interact with isocitrate dehydrogenase (IDH) proteins (49–51). IDH proteins form dimers or tetramers and catalyze the oxidative decarboxylation of isocitrate to α-ketoglutarate. Intriguingly, *IDH1* and *IDH2* are prominent mutated genes in diffuse glioma (52, 53). Furthermore, the gene encoding electron transfer flavoprotein α subunit (ETFA), which also interacts with IDH proteins (51), also comprises a glioma susceptibility locus (29). Thus, a cluster containing LRIG1, GLRX3, RAB4A, IDH proteins, and ETFA may comprise a functional subnetwork that is etiologically relevant in gliomagenesis.

A limitation of the current study is the fact that we were unable to confirm the shRNA-mediated decrease in protein levels in a systematic manner. The main reason for this shortcoming was the lack of suitable reagents for many of the targets (*i.e.* primarily, the lack of specific antibodies that were sufficiently sensitive to recognize endogenous protein levels in HEK293 cells). Therefore, the negative results should be interpreted cautiously. Furthermore, LRIG1 may have functions that are distinct from its regulation of RTK expression levels (14, 54, 55), which were not investigated in the current study. It is also important to note that the current study was unbiased and exploratory in nature rather than being confirmatory or mechanistically oriented. Therefore, further studies are needed to validate the interactions suggested herein and to elucidate the molecular mechanisms involved.

In summary, the results presented here delineate a proposed functional LRIG1 protein interaction network that impinges on the regulation of growth factor signaling and PDGFRA expression levels. Many of the described LRIG1 interactors were not previously anticipated to be regulators of LRIG1 function and therefore will provide novel leads in the quest to understand the molecular function of LRIG1 and its association with tumor suppression.

## Experimental procedures

### Yeast two-hybrid screen

The yeast two-hybrid screen was performed by Dualsystems Biotech AG (Zürich, Switzerland). The bait vector was created by subcloning cDNA encoding the entire cytosolic tail of LRIG1, corresponding to amino acids 816–1093 of GenBank^TM^ entry AF381545, into a *pLexA-DIR* vector (Dualsystems Biotech AG). The bait construct was transformed into the yeast strain DSY-1 (*MATa his3*Δ*200 trp1-901 leu2-3,112 ade2 LYS2::(lexAop)4-HIS3 URA3::(lexAop)8-lacZ GAL4*) using standard procedures (56). Correct expression of the bait was verified by Western blotting of cell extracts using a mouse monoclonal antibody directed against the LexA domain (Santa Cruz Biotechnology, Inc., Dallas, TX). The absence of self-activation was verified by co-transformation of the bait together with a control prey and selection on minimal medium lacking the amino acids tryptophan, leucine, and histidine (selective medium). For the yeast two-hybrid screen, DSY-1 cells were co-transformed with the bait together with a human adult whole-brain cDNA library. Positive transformants were tested for β-galactosidase activity using a filter assay (57). Library plasmids were isolated from positive clones and assayed in a bait dependence test with (i) the bait plasmid and (ii) a control bait encoding a LexA-laminC fusion protein using a mating strategy (58). The identity of positive interactors was determined by DNA sequencing.

### Plasmids and shRNAs

The expression vector encoding Myc-tagged LRIG1 (11) was kindly provided by Colleen Sweeney (University of California Davis, Sacramento, CA); pcDNA3.1 was obtained from Thermo Fisher Scientific (Gothenburg, Sweden), and pcDNA3-PDGFRα was a kind gift from Carl-Henrik Heldin (Ludwig Institute, Uppsala, Sweden). Bacterial glycerol stocks for five different shRNAs targeting EGFR and each of the assessed LRIG1 interactors were purchased from Sigma-Aldrich Sweden AB (Stockholm, Sweden) (Table S1). A GenElute HP plasmid miniprep kit (Sigma-Aldrich Sweden AB, PLN350) and a Nucleobond Xtra Midi/Maxi kit (Macherey-Nagel, 740410.50) were used to generate mini- and midi-preparations of each plasmid, respectively.

### Cell culture and transfections

The HEK293 cell line was obtained from the European Collection of Authenticated Cell Cultures (Salisbury, UK; 85120602) and authenticated using the DNA-profiling method (Leibniz-Institute DSMZ, Braunschweig, Germany). LRIG1-null HEK293T cells were generated through sequential CRISPR/CRISPR-associated protein-9 nuclease (Cas9)–mediated ablation of the entire *LRIG1* gene from HEK293T cells obtained from American Type Culture Collection (Manassas, VA). First, HEK293T cells were co-transfected with Cas9 and the guide RNAs GAGCGACTGATACTCCACAT and CACTTGCGCTGGGGACTCGC. From this transfection, one clone, clone 32, in which one *LRIG1* allele was deleted, was identified through genomic DNA sequencing. Clone 32 was then co-transfected with Cas9 and the guide RNAs AGCTGCGAACTCCGCCGATT and TACTGGGCTTCCGCGCGCTC. One clone, clone 3, was isolated from this transfection. Both LRIG1 alleles were completely deleted in clone 3 (*i.e.* exons 1–19 were biallelically deleted, as confirmed by genomic Sanger DNA sequencing). All cells were cultivated in DMEM (Sigma-Aldrich Sweden AB, D5796) supplemented with 10% fetal bovine serum (FBS) and 50 μg/ml gentamycin at 37 °C in a humidified 5% CO_2_ atmosphere. A MycoAlert mycoplasma detection kit (Lonza-BioNordica AB, Stockholm, Sweden, LT07) or a PCR mycoplasma detection set (Takara Bio Europe, Saint-Germain-en-Laye, France, 6601) was used for mycoplasma testing. Both the HEK293 and HEK293T LRIG1-null cells used in the present study were mycoplasma-free. X-tremeGENE HP DNA transfection reagent (Sigma-Aldrich Sweden AB, XTGHP-RO) was used to transfect HEK293 and LRIG1-null HEK293T cells cultured in 6-well plates (BD PureCoat amine cell culture plate, 356721) or on poly-d-lysine–coated glass slides (for confocal microscopy), according to the manufacturer's instructions, with a reagent/DNA ratio of 3:1. After the optimization experiments, the resulting triple co-transfection protocol included 0.5 μg of the *LRIG1* vector, 0.5 μg of the *PDGFRA* vector, and 1.25 μg of *shRNA* vector(s). When shRNA mixtures were used, cells were transfected with 250 ng of each of the five different shRNA plasmids.

### Antibodies

The primary antibodies used in this study included rabbit polyclonal anti-PDGFRα antibody (Santa Cruz Biotechnology, SC338, lots E2015 and E1115) at a 1:1,000 dilution; rabbit monoclonal anti-EGFR antibody (Cell Signaling Technology, Leiden, The Netherlands, 4267, lot 11) at a 1:1,000 dilution; mouse monoclonal anti-c-Myc antibody (Abcam, Cambridge, UK, ab32, lots GR178393, GR255064, and GR206680) at a 1:1,000 dilution for Western blotting and 1:200 for confocal microscopy; rabbit monoclonal anti-transferrin receptor antibody (Abcam, ab109259, lot GR GR84707) at a 1:5,000 dilution; rabbit monoclonal anti-PON2 antibody (Abcam, ab183710, lots GR153715-4 and GR153715-6) at a 1:20,000 dilution for Western blotting and 1:320 for confocal microscopy; rabbit monoclonal anti-Rab4 antibody (Abcam, ab108974, lot GR56968-5) at a 1:2,000 dilution; mouse monoclonal anti-actin antibody (Abcam, ab3280, lots GR269159-1, GR297708-1, and GR258587-1) at a 1:1,000 dilution; and mouse monoclonal anti-EGFR antibody (Dako, Glostrup, Denmark (now Agilent Technologies, Santa Clara, CA, USA), M3563, lot 0087E) at a 1:50 dilution. The secondary antibodies included horseradish peroxidase (HRP)-conjugated anti-whole-rabbit IgG antibody (GE Healthcare, Uppsala, Sweden, NA934) at a 1:20,000 dilution, HRP-conjugated anti-mouse IgG F(ab)2 fragment antibody (GE Healthcare, NA9310) at a 1:20,000 dilution, IRDye 680RD–conjugated goat anti-rabbit IgG (H + L) antibody and IRDye 800CW-conjugated goat anti-mouse IgG (H + L) antibody (LI-COR Biotechnology-UK Ltd., Cambridge, UK, 68071 and 32210, respectively) at a 1:20,000 dilution for Western blotting, and a highly cross-absorbed goat anti-mouse IgG (H+L) secondary antibody conjugated to Alexa Fluor 488 (Thermo Fisher Scientific, A11029) at a 1:500 dilution for FACS analysis and confocal microscopy and conjugated to Alexa Fluor 647 at a 1:500 dilution for confocal microscopy.

### Western blotting

Four days after transfection, the cells were lysed in ice-cold cell extraction buffer (Thermo Fisher Scientific, FNN0011) supplemented with EDTA-free protease inhibitor mixture (Sigma-Aldrich Sweden AB, 11873580001) and 1 mm phenylmethylsulfonyl fluoride. Samples were denatured with NuPAGE-LDS sample buffer (Thermo Fisher Scientific, NP0007) supplemented with NuPAGE sample-reducing agent (Thermo Fisher Scientific, NP0009) at 100 °C for 5 min, and then separated on a NuPAGE Novex 3–8% Tris acetate gel (Thermo Fisher Scientific, EA0375BOX) under reducing conditions by adding NuPAGE antioxidant (Thermo Fisher Scientific, NP0005). PON2 and RAB4A proteins were separated on a NuPAGE Novex 10% BisTris gel (Thermo Fisher Scientific, NP0302BOX). The separated proteins were transferred to polyvinylidene difluoride (Thermo Fisher Scientific, LC2002) or nitrocellulose (Bio-Rad Laboratories AB, Solna, Sweden, 170-4270) membranes using a Trans-Blot Turbo Blotting System (Bio-Rad Laboratories AB). The blot was blocked with 5 or 2% nonfat dry milk in TBST (200 mm Tris, pH 7.4, 150 mm NaCl, 0.1% Tween 20) and then incubated with primary antibodies diluted in 2.5 or 1% nonfat dry milk in TBST overnight at 4 °C or for 45 min at room temperature. For PON2 and RAB4A, Odyssey blocking buffer was used (LI-COR Biotechnology, 927-40000), and primary antibodies were diluted in this buffer. HRP-conjugated secondary antibodies and an Amersham Biosciences ECL Select Western blotting detection reagent kit (GE Healthcare, RPN2235) were used to detect the proteins. Images were acquired and analyzed with a ChemiDoc Touch Imaging System (Bio-Rad Laboratories AB) and Image Lab software (version 5.2.1). For PON2 and RAB4A, fluorescent secondary antibodies were used, and the signals were obtained and quantified using an Odyssey CLx imager (LI-COR Biotechnology) and Image Studio Lite software (version 5.2). Actin was used as an internal loading control. All Western blots for PDGFRA, with the exception of the experiment shown in Fig. 1*A*, were performed in a blinded manner in which the investigator was not aware of the identities of the samples.

### Flow cytometry

HEK293 cells cultured in 6-well plates were detached by incubating cells with 200 μl of Accutase per well (Sigma-Aldrich Sweden AB, A6964) for 10 min. The detached cells were centrifuged at 400 × *g* for 5 min, washed with cold PBS, and incubated with primary antibody diluted in FACS buffer (3% FBS and 0.02% NaN_3_ in PBS) on ice for 30 min. Thereafter, cells were washed twice and then incubated with the fluorescent secondary antibody on ice for 30 min. After two washes, cells were resuspended in FACS buffer supplemented with propidium iodide (0.1 μg/ml) to label the dead cells. The labeled cells were analyzed using a BD Accuri C6 flow cytometer; data from 10,000 cells were acquired for each sample. The data were corrected by a 5% subtraction and analyzed using FlowJo software (version 10).

### In silico analyses

The RNAseq counts data from all solid tumors in the TCGA database (https://gdc.cancer.gov,^3^ May 15, 2017) were downloaded. We also downloaded normalized counts data using the fragments per kilobase of transcript per million mapped reads upper quartile (FPKM-UQ) method to calculate correlations. We only analyzed samples marked as primary solid tumors or solid tissue normal. Correlations between *LRIG1* expression and levels of the LRIG1 interactor transcripts were calculated in each tissue, but only tissues with more than five samples were considered, using the FPKM-UQ data and Spearman's ρ. These calculations were performed with the package edgeR (59) using the R programming language.

### Quantitative real-time RT-PCR

A PARIS kit (Thermo Fisher Scientific, AM1921) was used to isolate RNA from HEK293 cells 2 days after transfection. After treating the isolated RNA with reagents from a TURBO DNA-free kit (Thermo Fisher Scientific, AM1907), a qScript One-Step quantitative RT-PCR kit (95057, Quanta Biosciences) was used to synthesize cDNA, and 40 ng of total RNA was used for PCR. A CFX96 system C1000 thermal cycler was used, and the RT-PCR parameters were as follows: 50 °C for 10 min and 95 °C for 5 min, followed by 45 cycles at 95 °C for 15 s and 60 °C for 30 s. The *ZBTB16* TaqMan gene expression assay (Hs00232313_m1) was purchased from Applied Biosystems; the *LRIG1* assay has been described previously (1). Data were normalized using *RN18S* as the reference gene, as described previously (1). Standard curves were prepared to evaluate RT-PCR amplification efficiencies. Data were analyzed using the 2^−ΔΔ*Ct*^ (Livak) method for relative gene expression analysis.

### Confocal laser-scanning microscopy

For confocal laser-scanning microscopy, HEK293 cells were grown on No. 1.5 glass coverslips coated with 25 μg/ml poly-d-lysine hydrobromide (Sigma-Aldrich Sweden AB), followed by transfection with the indicated plasmids. Forty-eight hours after transfection, cells were fixed with 4% paraformaldehyde as described previously (15) followed by permeabilization and blocking with PBS containing 5% FBS and 0.05% Tween 20. Cells were incubated with primary and secondary antibodies in PBS containing 5% FBS and 0.05% Tween 20 for 1 h at ambient temperature with washes between the incubations. Prolong Gold (Thermo Fisher Scientific, P36930) with DAPI was used to mount the samples and counterstain nuclei; 3D images were acquired at ambient temperature with a Zeiss LSM 710 confocal microscope equipped with a plan-apochromat 63 × 1.4 numerical aperture objective controlled by ZEN 201 SP1 software (Carl Zeiss AB, Stockholm, Sweden). Pixel size was set according to the Nyquist criterion. For quantification of PON2 expression, the fields of view were exclusively selected based on the DAPI nuclear staining. The mean total intensity was calculated using IMARIS software (Bitplane AG, Zürich, Switzerland). At least 500 cells were quantified. *z*-Stacks were displayed as maximum *z*-projections, and brightness and contrast were adjusted (identically for all images) using ZEN 210 SP1 software. For the co-localization analysis, *z*-stacks with 0.6-μm step sizes were acquired. To quantify the degree of LRIG1 and PON2 co-localization, 3D images were preprocessed, and cellular regions of interest were segmented using ImageJ (60). The cellular regions of interest for each channel were used to quantify the Pearson (M1 and M2) coefficients using ImageJ with the JACoP plugin (61).

### Statistical analyses

All experiments, with the exception of the yeast two-hybrid screen, were performed at least three independent times. The data are expressed as the mean ± S.D. of the different independent biological replicates. Unpaired two-tailed Student's *t* test was used to determine the significance of differences, and *p* < 0.01 (experiments shown in Fig. 2) or *p* < 0.05 (all other experiments) was considered significant.

## Author contributions

M. F. designed the study; performed Western blotting, quantitative RT-PCR, and FACS experiments; analyzed the results; and drafted the manuscript. C. Herdenberg performed the TCGA analyses. C. Holmlund performed the confocal microscopy experiments. R. H. conceived the study and drafted the manuscript. H. H. conceived, designed, and coordinated the study; interpreted the results; and wrote the paper. All authors reviewed the results and approved the final version of the manuscript.

## Supplementary Material

Supporting Information

## Footnotes

3 Please note that the JBC is not responsible for the long-term archiving and maintenance of this site or any other third party hosted site.

## Footnotes

2 The abbreviations used are:

RTK: receptor tyrosine kinase
EGFR: epidermal growth factor receptor
RET: rearranged during transfection
PDGFRA: platelet-derived growth factor receptor α
BioPlex: biophysical interactions of ORFeome-based complexes
HEK: human embryonic kidney
TCGA: The Cancer Genome Atlas
TFRC: transferrin receptor
IDH: isocitrate dehydrogenase
ETFA: electron transfer flavoprotein α subunit
HRP: horseradish peroxidase
BisTris: 2-[bis(2-hydroxyethyl)amino]-2-(hydroxymethyl)propane-1,3-diol
FPKM-UQ: fragments per kilobase of transcript per million mapped reads upper quartile
FBS: fetal bovine serum
FGF: fibroblast growth factor
DAPI: 4′,6-diamidino-2-phenylindole.
