# Supplementary material for: A protein interaction network centered on leucine-rich repeats and immunoglobulin-like domains 1 (LRIG1) regulates growth factor receptors
Source: J Biol Chem. 2018 Jan 9;293(9):3421–35. doi: 10.1074/jbc.M117.807487 (PMC5836135; doi:10.1074/jbc.M117.807487)
Supplement: Supporting Information [file supp_293_9_3421__index.html]

A protein interaction network centered on leucine-rich repeats and immunoglobulin-like domains 1 (LRIG1) regulates growth factor receptors — A protein interaction network centered on leucine-rich repeats and immunoglobulin-like domains 1 (LRIG1) regulates growth factor receptors — LRIG1 interactome — Supporting Information 

# A protein interaction network centered on leucine-rich repeats and immunoglobulin-like domains 1 (LRIG1) regulates growth factor receptors

## Supporting Information

- Supplementary Table 1 (.docx, 41 KB) - ShRNAs characteristics used in the study
