# Supplementary material for: A protein interaction network centered on leucine-rich repeats and immunoglobulin-like domains 1 (LRIG1) regulates growth factor receptors
Source: J Biol Chem. 2018 Jan 9;293(9):3421–35. doi: 10.1074/jbc.M117.807487 (PMC5836135; doi:10.1074/jbc.M117.807487)
Supplement: Supporting Information [file 10.1074_M117.807487_jbc.M117.807487-1.docx]

**A protein interaction network centered on leucine-rich repeats and immunoglobulin-like domains 1 (LRIG1) regulates growth factor receptors**

Mahmood Faraz^1^, Carl Herdenberg^1^, Camilla Holmlund^1^, Roger Henriksson^1^, and Håkan Hedman^1,2^

^1^Oncology Research Laboratory, Department of Radiation Sciences, Umeå University, SE-90187 Umeå, Sweden.

^2^ To whom correspondence should be addressed. Tel.: 0046-90-785 2881; Fax: 0046-90-785 2031; E-mail: [hakan.hedman@umu.se](mailto:hakan.hedman@umu.se)

**Supplemental data:**

**Table S1.**

| **Clone ID**  **Table S1. ShRNAs characteristics used in the study.** | **shRNA** | **Oligo Sequence** | **Symbol** | **Gene ID** | **Validated in cell line** |
| --- | --- | --- | --- | --- | --- |
| TRCN0000001423 | A1 | CCGGCCAGCCTCATATAAGAAGAAACTCGAGTTTCTTCTTATATGAGGCTGGTTTTT | PDGFRA | 5156 |  |
| TRCN0000001425 | A2 | CCGGCGGTGAAAGACAGTGGAGATTCTCGAGAATCTCCACTGTCTTTCACCGTTTTT | PDGFRA | 5156 | MCH58 |
| TRCN0000194855 | 2A11 | CCGGCCCTCTGAAATAATGGGATTACTCGAGTAATCCCATTATTTCAGAGGGTTTTTTG | PDGFRA | 5156 | MCH58 |
| TRCN0000196928 | 2A12 | CCGGGCTAGCAATTGCGACCTTAATCTCGAGATTAAGGTCGCAATTGCTAGCTTTTTTG | PDGFRA | 5156 | MCH58 |
| TRCN0000195132 | 2B1 | CCGGCTACTACTGTTATCAGTAATGCTCGAGCATTACTGATAACAGTAGTAGTTTTTTG | PDGFRA | 5156 | MCH58 |
| TRCN0000002873 | A3 | CCGGGCTGCTTTCATCGTCACACAACTCGAGTTGTGTGACGATGAAAGCAGCTTTTT | PTPRK | 5796 |  |
| TRCN0000002874 | A4 | CCGGGCCCAGACTAAGAACATCAATCTCGAGATTGATGTTCTTAGTCTGGGCTTTTT | PTPRK | 5796 | A3 |
| TRCN0000002875 | A5 | CCGGCCGGCGAGTCAAGTTATCAAACTCGAGTTTGATAACTTGACTCGCCGGTTTTT | PTPRK | 5796 |  |
| TRCN0000002876 | A6 | CCGGCATACTTTCTACGTGGCATTTCTCGAGAAATGCCACGTAGAAAGTATGTTTTT | PTPRK | 5796 |  |
| TRCN0000002877 | A7 | CCGGGCTCCAACTTTACCTGACTATCTCGAGATAGTCAGGTAAAGTTGGAGCTTTTT | PTPRK | 5796 |  |
| TRCN0000004457 | A12 | CCGGCCTGAATGATGTGTCTCTGCACTCGAGTGCAGAGACACATCATTCAGGTTTTT | SCRIB | 23513 | A549 |
| TRCN0000004458 | B1 | CCGGCTGGCCTGTGACTAACTAACTCTCGAGAGTTAGTTAGTCACAGGCCAGTTTTT | SCRIB | 23513 |  |
| TRCN0000004459 | B2 | CCGGCAGCTATCCATCCTAAAGGTACTCGAGTACCTTTAGGATGGATAGCTGTTTTT | SCRIB | 23513 |  |
| TRCN0000004460 | B3 | CCGGGCATATTCATCTCTCGGGTGTCTCGAGACACCCGAGAGATGAATATGCTTTTT | SCRIB | 23513 |  |
| TRCN0000004461 | B4 | CCGGGTCCCTGTCATTTCTGGTCAACTCGAGTTGACCAGAAATGACAGGGACTTTTT | SCRIB | 23513 | A549 |
| TRCN0000007990 | B11 | CCGGGCATGGAATCTGATGTATGATCTCGAGATCATACATCAGATTCCATGCTTTTT | RAB4A | 5867 | A3, HEK293* |
| TRCN0000007992 | B12 | CCGGCGAGAAACCTACAATGCGCTTCTCGAGAAGCGCATTGTAGGTTTCTCGTTTTT | RAB4A | 5867 | A3 |
| TRCN0000007993 | C1 | CCGGTCAGGTATTCAGTACGGAGATCTCGAGATCTCCGTACTGAATACCTGATTTTT | RAB4A | 5867 | 293T/17 |
| TRCN0000231997 | 2B12 | CCGGTGCTTACTTCATCAGTTTATTCTCGAGAATAAACTGATGAAGTAAGCATTTTTG | RAB4A | 5867 | 293T/17, HEK293* |
| TRCN0000011217 | 2D7 | CCGGAGGAGTGGAATTTGGTTCAAACTCGAGTTTGAACCAAATTCCACTCCTTTTTT | RAB4A | 5867 | A3 |
| TRCN0000012938 | C2 | CCGGCCACCGCAATAGTCAACACTACTCGAGTAGTGTTGACTATTGCGGTGGTTTTT | ZBTB16 | 7704 |  |
| TRCN0000012939 | C3 | CCGGGAATGCACTTACTGGCTCATTCTCGAGAATGAGCCAGTAAGTGCATTCTTTTT | ZBTB16 | 7704 |  |
| TRCN0000012940 | C4 | CCGGGTGGACAGTTTGATGACCATACTCGAGTATGGTCATCAAACTGTCCACTTTTT | ZBTB16 | 7704 |  |
| TRCN0000012942 | C5 | CCGGGCAATAGTCAACACTATACTTCTCGAGAAGTATAGTGTTGACTATTGCTTTTT | ZBTB16 | 7704 |  |
| TRCN0000367816 | 2D1 | CCGGATCAGCTGGAGACGCACTATACTCGAGTATAGTGCGTCTCCAGCTGATTTTTTG | ZBTB16 | 7704 |  |
| TRCN0000035615 | C6 | CCGGCTGGAAGTTCATTCGCGATTTCTCGAGAAATCGCGAATGAACTTCCAGTTTTTG | GAL3ST1 | 9514 | HEK293* |
| TRCN0000035616 | C7 | CCGGCGAGTCCTCCTTCCACTACTTCTCGAGAAGTAGTGGAAGGAGGACTCGTTTTTG | GAL3ST1 | 9514 | HEK293* |
| TRCN0000035617 | C8 | CCGGCCTTCAAGAGTACTTCGACGACTCGAGTCGTCGAAGTACTCTTGAAGGTTTTTG | GAL3ST1 | 9514 |  |
| TRCN0000035618 | C9 | CCGGGCTCTTCACTAGTTTCCTGCTCTCGAGAGCAGGAAACTAGTGAAGAGCTTTTTG | GAL3ST1 | 9514 |  |
| TRCN0000035614 | 2D8 | CCGGGCGCAACATCGTGTTCTTGAACTCGAGTTCAAGAACACGATGTTGCGCTTTTTG | GAL3ST1 | 9514 | HEK293* |
| TRCN0000051643 | D4 | CCGGGCACATTTCTATGCCACAAATCTCGAGATTTGTGGCATAGAAATGTGCTTTTTG | PON2 | 5445 | A549 |
| TRCN0000051644 | D5 | CCGGGCACTCAGAAATCGACTTAAACTCGAGTTTAAGTCGATTTCTGAGTGCTTTTTG | PON2 | 5445 | A549, HEK293* |
| TRCN0000051645 | D6 | CCGGCCACTACTTCTCTGATCCTTTCTCGAGAAAGGATCAGAGAAGTAGTGGTTTTTG | PON2 | 5445 |  |
| TRCN0000051646 | D7 | CCGGGACTACAGTTTATGCCAACAACTCGAGTTGTTGGCATAAACTGTAGTCTTTTTG | PON2 | 5445 | A549, HEK293* |
| TRCN0000051647 | D8 | CCGGGCTGCTCATAGGCACTTTATACTCGAGTATAAAGTGCCTATGAGCAGCTTTTTG | PON2 | 5445 | A549, HEK293* |
| TRCN0000051898 | D9 | CCGGCAGATCATTGTGGCCCTCAAACTCGAGTTTGAGGGCCACAATGATCTGTTTTTG | CANT1 | 124583 |  |
| TRCN0000051900 | D10 | CCGGCAAGATCGGAAGCGTGAAATACTCGAGTATTTCACGCTTCCGATCTTGTTTTTG | CANT1 | 124583 |  |
| TRCN0000051901 | D11 | CCGGGAAGGTGATCCTGACGTTCTTCTCGAGAAGAACGTCAGGATCACCTTCTTTTTG | CANT1 | 124583 |  |
| TRCN0000051902 | 2D9 | CCGGGAACTGGGTGTCCAACTACAACTCGAGTTGTAGTTGGACACCCAGTTCTTTTTG | CANT1 | 124583 |  |
| TRCN0000051899 | 2D10 | CCGGCGGCTGGGATTCGGTATCGAACTCGAGTTCGATACCGAATCCCAGCCGTTTTTG | CANT1 | 124583 |  |
| TRCN0000057293 | E4 | CCGGGCCAGGGACACCGCACAGATTCTCGAGAATCTGTGCGGTGTCCCTGGCTTTTTG | HLA-E | 3133 |  |
| TRCN0000057294 | E5 | CCGGCGGCAAGGATTATCTCACCCTCTCGAGAGGGTGAGATAATCCTTGCCGTTTTTG | HLA-E | 3133 |  |
| TRCN0000057295 | E6 | CCGGGCTCCACAAATACCTGGAGAACTCGAGTTCTCCAGGTATTTGTGGAGCTTTTTG | HLA-E | 3133 |  |
| TRCN0000057296 | E7 | CCGGCCTTGAAGTATTTCCACACTTCTCGAGAAGTGTGGAAATACTTCAAGGTTTTTG | HLA-E | 3133 |  |
| TRCN0000423889 | 2D5 | CCGGCACCTCTGTGTCTACCATGACCTCGAGGTCATGGTAGACACAGAGGTGTTTTTTG | HLA-E | 3133 |  |
| TRCN0000057308 | E8 | CCGGGCTTTACAAAGCTGGCAATATCTCGAGATATTGCCAGCTTTGTAAAGCTTTTTG | HLA-DRA | 3122 |  |
| TRCN0000057310 | E9 | CCGGCTGACCAATCAGGCGAGTTTACTCGAGTAAACTCGCCTGATTGGTCAGTTTTTG | HLA-DRA | 3122 |  |
| TRCN0000057311 | E10 | CCGGCAGGCCGAGTTCTATCTGAATCTCGAGATTCAGATAGAACTCGGCCTGTTTTTG | HLA-DRA | 3122 |  |
| TRCN0000057312 | E11 | CCGGCCATCTTCATCATCAAGGGATCTCGAGATCCCTTGATGATGAAGATGGTTTTTG | HLA-DRA | 3122 |  |
| TRCN0000413580 | 2E6 | CCGGAGGAATCATGGGCTATCAAAGCTCGAGCTTTGATAGCCCATGATTCCTTTTTTTG | HLA-DRA | 3122 |  |
| TRCN0000063453 | F1 | CCGGCCAGTCTATTTATGACAAGAACTCGAGTTCTTGTCATAAATAGACTGGTTTTTG | SCARA3 | 51435 |  |
| TRCN0000063454 | F2 | CCGGCGAGATTGAAATTGGCACCATCTCGAGATGGTGCCAATTTCAATCTCGTTTTTG | SCARA3 | 51435 |  |
| TRCN0000063455 | F3 | CCGGGCTGCCAGAAGAACCTATCTTCTCGAGAAGATAGGTTCTTCTGGCAGCTTTTTG | SCARA3 | 51435 |  |
| TRCN0000063457 | F4 | CCGGCTTCGCAATGTCACCATCCTACTCGAGTAGGATGGTGACATTGCGAAGTTTTTG | SCARA3 | 51435 | A549 |
| TRCN0000323047 | 2C8 | CCGGACAGCTGTGGTCCTCTGATTCCTCGAGGAATCAGAGGACCACAGCTGTTTTTTG | SCARA3 | 51435 |  |
| TRCN0000064663 | F5 | CCGGCCCTCAAGTTTCATTTGTGAACTCGAGTTCACAAATGAAACTTGAGGGTTTTTG | GLRX3 | 10539 | A549 |
| TRCN0000064664 | F6 | CCGGGCTCTTTATGAAAGGAAACAACTCGAGTTGTTTCCTTTCATAAAGAGCTTTTTG | GLRX3 | 10539 | A549 |
| TRCN0000064665 | F7 | CCGGCCAGCGCTAATGAACATCTTACTCGAGTAAGATGTTCATTAGCGCTGGTTTTTG | GLRX3 | 10539 | A549, HEK293* |
| TRCN0000064666 | F8 | CCGGCCTACCTATCCTCAGCTCTATCTCGAGATAGAGCTGAGGATAGGTAGGTTTTTG | GLRX3 | 10539 | A549 |
| TRCN0000064667 | F9 | CCGGGAACGAAGTTATGGCAGAGTTCTCGAGAACTCTGCCATAACTTCGTTCTTTTTG | GLRX3 | 10539 | A549, HEK293* |
| TRCN0000116637 | F11 | CCGGGCCACCTTCATTGGGAATAATCTCGAGATTATTCCCAATGAAGGTGGCTTTTTG | TUBB8 | 347688 |  |
| TRCN0000116638 | F12 | CCGGCCAGCAGATGTTTGATGCTAACTCGAGTTAGCATCAAACATCTGCTGGTTTTTG | TUBB8 | 347688 |  |
| TRCN0000116639 | G1 | CCGGGTACCCTTCTGCTCAGTAAGACTCGAGTCTTACTGAGCAGAAGGGTACTTTTTG | TUBB8 | 347688 |  |
| TRCN0000116640 | G2 | CCGGCCCAGCAGATGTTTGATGCTACTCGAGTAGCATCAAACATCTGCTGGGTTTTTG | TUBB8 | 347688 |  |
| TRCN0000116641 | G3 | CCGGGCCGTGAACATGGTCCCGTTTCTCGAGAAACGGGACCATGTTCACGGCTTTTTG | TUBB8 | 347688 |  |
| TRCN0000118062 | G4 | CCGGGCCTCTATCATAGTCAGCAATCTCGAGATTGCTGACTATGATAGAGGCTTTTTG | GML | 2765 |  |
| TRCN0000118063 | G5 | CCGGCGTATCATATTAGGCGCTGTACTCGAGTACAGCGCCTAATATGATACGTTTTTG | GML | 2765 |  |
| TRCN0000118064 | G6 | CCGGCGCATAAATTCTCGTGAACTACTCGAGTAGTTCACGAGAATTTATGCGTTTTTG | GML | 2765 |  |
| TRCN0000118065 | G7 | CCGGCCGTATCATATTAGGCGCTGTCTCGAGACAGCGCCTAATATGATACGGTTTTTG | GML | 2765 |  |
| TRCN0000118066 | G8 | CCGGGTTGTTGTAATAGCATGGTTTCTCGAGAAACCATGCTATTACAACAACTTTTTG | GML | 2765 |  |
| TRCN0000039633 | C10 | CCGGGCTGAGAATGTGGAATACCTACTCGAGTAGGTATTCCACATTCTCAGCTTTTTG | EGFR | 1956 | A549 |
| TRCN0000121067 | G9 | CCGGGCTGCTCTGAAATCTCCTTTACTCGAGTAAAGGAGATTTCAGAGCAGCTTTTTG | EGFR | 1956 | A-431 |
| TRCN0000121068 | G10 | CCGGGCCACAAAGCAGTGAATTTATCTCGAGATAAATTCACTGCTTTGTGGCTTTTTG | EGFR | 1956 | A549 |
| TRCN0000010329 | 2D11 | CCGGAGAATGTGGAATACCTAAGGCTCGAGCCTTAGGTATTCCACATTCTCTTTTTG | EGFR | 1956 | A549 |
| TRCN0000121069 | 2E3 | CCGGCGCAAAGTGTGTAACGGAATACTCGAGTATTCCGTTACACACTTTGCGTTTTTG | EGFR | 1956 | A-431 |
| TRCN0000134783 | G11 | CCGGCGAATGAAACAGAGTCAAGATCTCGAGATCTTGACTCTGTTTCATTCGTTTTTTG | MTFR1L | 56181 | MCH58 |
| TRCN0000138298 | G12 | CCGGCGCAGCCAGATTGCAAAGATACTCGAGTATCTTTGCAATCTGGCTGCGTTTTTTG | MTFR1L | 56181 | MCH58 |
| TRCN0000138856 | H1 | CCGGGCGGATGAAGAGGAGACATATCTCGAGATATGTCTCCTCTTCATCCGCTTTTTTG | MTFR1L | 56181 | MCH58 |
| TRCN0000134118 | H2 | CCGGCAGGAAGTTCAAATGTCTCTTCTCGAGAAGAGACATTTGAACTTCCTGTTTTTTG | MTFR1L | 56181 | MCH58 |
| TRCN0000138499 | H3 | CCGGCGGCTTCATTAACGCCAGATTCTCGAGAATCTGGCGTTAATGAAGCCGTTTTTTG | MTFR1L | 56181 | MCH58 |
| TRCN0000129881 | H4 | CCGGGACCTTTGAGACATTACACAACTCGAGTTGTGTAATGTCTCAAAGGTCTTTTTTG | CNPY3 | 10695 | A549 |
| TRCN0000127844 | H5 | CCGGGTCAGAGACCTTTGAGACATTCTCGAGAATGTCTCAAAGGTCTCTGACTTTTTTG | CNPY3 | 10695 | A549 |
| TRCN0000130772 | H6 | CCGGGAGACATTACACAACCTGGTACTCGAGTACCAGGTTGTGTAATGTCTCTTTTTTG | CNPY3 | 10695 | A549 |
| TRCN0000129597 | H7 | CCGGCCTGGATTATAGCCTGCACAACTCGAGTTGTGCAGGCTATAATCCAGGTTTTTTG | CNPY3 | 10695 |  |
| TRCN0000422180 | 2D6 | CCGGTCTGCCAAGGAAAGACACAAGCTCGAGCTTGTGTCTTTCCTTGGCAGATTTTTTG | CNPY3 | 10695 |  |
| TRCN0000160862 | H8 | CCGGGCTCCTAGAGATGAACTCTATCTCGAGATAGAGTTCATCTCTAGGAGCTTTTTTG | CNPY4 | 245812 |  |
| TRCN0000159014 | H9 | CCGGGAAGAGGCCTTAGAGAATTTACTCGAGTAAATTCTCTAAGGCCTCTTCTTTTTTG | CNPY4 | 245812 |  |
| TRCN0000163917 | H10 | CCGGCCAGCAAATGCGAAGTGTGTACTCGAGTACACACTTCGCATTTGCTGGTTTTTTG | CNPY4 | 245812 |  |
| TRCN0000135317 | H11 | CCGGCTTACAGCGTTTCAGAGACAACTCGAGTTGTCTCTGAAACGCTGTAAGTTTTTTG | CNPY4 | 245812 |  |
| TRCN0000136783 | 2B10 | CCGGCATTGTGGGAGACTGGTACTTCTCGAGAAGTACCAGTCTCCCACAATGTTTTTTG | CNPY4 | 245812 |  |
| TRCN0000151905 | 2A1 | CCGGCTGGGAATGATGCTCATATAACTCGAGTTATATGAGCATCATTCCCAGTTTTTTG | LRIG3 | 121227 |  |
| TRCN0000150765 | 2A2 | CCGGGCAGGAAGTATTTCAGCAAATCTCGAGATTTGCTGAAATACTTCCTGCTTTTTTG | LRIG3 | 121227 |  |
| TRCN0000154114 | 2A3 | CCGGCGACTGATACTCCAAGGAAATCTCGAGATTTCCTTGGAGTATCAGTCGTTTTTTG | LRIG3 | 121227 |  |
| TRCN0000151792 | 2B8 | CCGGCCAGCAGATATTCCTAGTTATCTCGAGATAACTAGGAATATCTGCTGGTTTTTTG | LRIG3 | 121227 |  |
| TRCN0000151855 | 2B9 | CCGGCGAGTAATTCTTTCATGGGTACTCGAGTACCCATGAAAGAATTACTCGTTTTTTG | LRIG3 | 121227 |  |
| TRCN0000154082 | 2A4 | CCGGCCATGCGTTGTTTGGTCTTATCTCGAGATAAGACCAAACAACGCATGGTTTTTTG | LRIG2 | 9860 |  |
| TRCN0000155196 | 2A5 | CCGGGCCATGCGTTGTTTGGTCTTACTCGAGTAAGACCAAACAACGCATGGCTTTTTTG | LRIG2 | 9860 | A549 |
| TRCN0000152310 | 2A6 | CCGGCATGCGTTGTTTGGTCTTATACTCGAGTATAAGACCAAACAACGCATGTTTTTTG | LRIG2 | 9860 |  |
| TRCN0000152196 | 2A7 | CCGGCCGAACTTGATTTGTCCTATACTCGAGTATAGGACAAATCAAGTTCGGTTTTTTG | LRIG2 | 9860 |  |
| TRCN0000151448 | 2B7 | CCGGCAGTCATAATCGGTTGTCTAACTCGAGTTAGACAACCGATTATGACTGTTTTTTG | LRIG2 | 9860 |  |
| TRCN0000072660 | F10 | CCGGGCCAGCAGAAATAAATAGAATCTCGAGATTCTATTTATTTCTGCTGGCTTTTTG | LRRC40 | 55631 | HeLa |
| TRCN0000344884 | 2C9 | CCGGATCACCTACCCGTTGGTATAACTCGAGTTATACCAACGGGTAGGTGATTTTTTG | LRRC40 | 55631 | HeLa |
| TRCN0000344883 | 2C10 | CCGGTTCCACCAGAGCTCGGTAATTCTCGAGAATTACCGAGCTCTGGTGGAATTTTTG | LRRC40 | 55631 | HeLa |
| TRCN0000344938 | 2C11 | CCGGACCTAAGCAACAATGATATTACTCGAGTAATATCATTGTTGCTTAGGTTTTTTG | LRRC40 | 55631 | HeLa |
| TRCN0000333593 | 2C12 | CCGGCGCAGTGTGTCTGGAGAATAACTCGAGTTATTCTCCAGACACACTGCGTTTTTG | LRRC40 | 55631 | HeLa |
| TRCN0000289919 | L11 | CCGGCCCTTTCTGACCGACAAGAAACTCGAGTTTCTTGTCGGTCAGAAAGGGTTTTTG | LRIG1 | 26018 | 293T/17 |
| TRCN0000289844 | L12 | CCGGGCCGGTTCTATTTCAGCTAATCTCGAGATTAGCTGAAATAGAACCGGCTTTTTG | LRIG1 | 26018 | 293T/17 |
| TRCN0000222556 | L13 | CCGGGCGGAGCCTAAACCTGAGTTACTCGAGTAACTCAGGTTTAGGCTCCGCTTTTTG | LRIG1 | 26018 |  |
| TRCN0000310218 | L14 | CCGGATCTGGACCATAACGAGATTTCTCGAGAAATCTCGTTATGGTCCAGATTTTTTG | LRIG1 | 26018 | 293T/17 |
| TRCN0000296342 | L15 | CCGGTCCACACGGACCGCCTATAAACTCGAGTTTATAGGCGGTCCGTGTGGATTTTTG | LRIG1 | 26018 | 293T/17 |

*Validated in-house through quantitative RT-PCR analyses of triple co-transfected HEK293 cells according to the protocol indicated in the Materials and Methods section.

S3
